# Effectiveness of sulphonylureas in the therapy of diabetes mellitus type 2 patients: an observational cohort study

**DOI:** 10.1186/s40200-016-0251-9

**Published:** 2016-08-02

**Authors:** Thomas Wilke, Sabrina Mueller, Antje Groth, Bjoern Berg, Niklas Hammar, Katherine Tsai, Andreas Fuchs, Stephanie Stephens, Ulf Maywald

**Affiliations:** 1 IPAM, University of Wismar, Alter Holzhafen 19, 23966 Wismar, Germany; 2 AstraZeneca R&D Mölndal, Pepparedsleden 1, Mölndal, 431 83 Sweden; 3 AstraZeneca R&D, 101 Orchard Ridge Drive, 2207K, Gaithersburg, MD 20878 USA; 4 AOK PLUS, Sternplatz 7, 01067 Dresden, Germany; 5 Pharmerit Eu York, Enterprise House, Innovation Way, YO10 5NQ York, UK

**Keywords:** Type 2 diabetes mellitus, Sulphonylureas, Antidiabetic therapy, Macrovascular event risk, Mortality risk for type 2 diabetes mellitus patients, T2DM-related hospitalizations

## Abstract

**Background:**

We compared all-cause mortality, major macrovascular events (MACE) and diabetes-related hospitalizations in T2DM-incident patients newly treated with metformin (MET) versus sulphonylureas (SU) monotherapy and in T2DM-prevalent patients newly treated with MET+SU versus MET+DPP4-inhibitor combination therapy.

**Methods:**

We analysed anonymized data obtained from a German health fund. Patients were included when they had started MET versus SU therapy or MET+SU versus MET+DPP4 therapy between 01/07/2010 and 31/12/2011. Observation started with the first MET/SU prescription or the first prescription of the second agent of a MET+SU/MET+DPP4 combination therapy. Follow-up time lasted until the end of data availability (a minimum of 12 months), death or therapy discontinuation.

**Results:**

In total, 434,291 T2DM-prevalent and 35,661 T2DM-incident patients were identified. Of the identified T2DM-incident patients, 904/7,874 started SU/MET monotherapy, respectively, with a mean age of 70.1/61.4 years (54.6/50.3 % female; Charlson Comorbidity Index (CCI) 1.4/2.2; 933/7,350 observed SU/MET patient years). 4,157/1,793 SU+MET/DPP4+MET therapy starters had a mean age of 68.1/62.2 years (53.4/50.8 % female; CCI 2.8/2.6; 4,556/1,752 observed SU+MET/ DPP4+MET patient years).

In a propensity score matched (PSM) comparison, the HRs (95 % CIs) associated with SU monotherapy compared to MET monotherapy exposure were 1.4 (0.9–2.3) for mortality, 1.4 (0.9–2.2) for MACE, 4.1 (1.5–10.9) for T2DM hospitalizations and 1.6 (1.2–2.3) for composite event risk. In a multivariable Cox regression model, SU monotherapy was associated with higher mortality (aHR 2.0; 1.5–2.6), higher MACE (aHR 1.3; 1.0–1.7) and higher T2DM hospitalizations (aHR 2.8; 1.8–4.4), which corresponded with a higher composite event risk (aHR 1.8; 1.5–2.1).

No significant differences in event rates were observed in the PSM comparison between DPP4+MET/SU+MET combination therapy starters and in the multivariable Cox regression analysis.

**Conclusions:**

Our results show that SU monotherapy may be associated with increased mortality, MACE and T2DM hospitalizations, compared to MET monotherapy. When considering SU therapy, the associated cardiovascular risk should also be taken into account.

**Electronic supplementary material:**

The online version of this article (doi:10.1186/s40200-016-0251-9) contains supplementary material, which is available to authorized users.

## Background

Amongst the most common chronic diseases, type 2 diabetes mellitus (T2DM) presents some of the greatest clinical and health economic challenges [1]. In addition to burdens directly associated with the underlying disease, T2DM patients have an increased frequency of micro- and macrovascular complications and hospitalizations as well as increased mortality rates [2–7].

The primary goal of diabetes treatment is to control blood glucose levels [8, 9]. If treatment with metformin (MET) is insufficient, treatment guidelines recommend second-line treatment with agents including sulphonylureas (SU), thiazolidinediones, alpha-glucosidase inhibitors, dipeptidyl peptidase-4 inhibitors (DPP4), basal insulin, SGLT-2 inhibitors and glucagon-like peptide-1 (GLP-1) receptor agonists [8, 9].

Previous observational studies have shown that a substantial number of T2DM patients receive SUs [10, 11]. In fact, in countries like Germany, public agencies frequently see SUs as a main comparator therapy when assessing the potential value and reimbursement price of new second-line T2DM treatment agents such as DPP4s or GLP1s [12–14]. That being said, findings from clinical trials and observational studies have also raised concerns about the effectiveness and safety of SU treatment, especially in terms of its association with risks of hypoglycaemic as well as macrovascular events [11, 15–19]. Specifically, a recent UK analysis concluded that both SU monotherapy (compared to MET monotherapy) and SU combination therapy with MET (compared to MET+DPP4 combination therapy) are associated with an increased macrovascular/mortality event risk [11, 19].

In this study, we assessed all-cause mortality, major macrovascular events (MACE) and diabetes-related hospitalizations in T2DM-incident patients newly treated with MET versus SU monotherapy and in T2DM-prevalent patients newly treated with MET+DPP4 versus MET+SU combination therapy.

## Methods

### T2DM samples

We used an anonymized dataset obtained from the German health fund AOK PLUS (2010–2012) which initially included all T2DM-prevalent patients [at least one outpatient or inpatient T2DM diagnosis (ICD-10 codes: E11.-) in 01/07/2010-31/12/2011] who were insured by this health fund for the entire study period. The dataset contained information on patient socio-demographics, outpatient prescriptions, diagnosis-associated outpatient visits to GPs and specialists, and finally inpatient treatment in hospitals.

All patients were followed from the moment they were enrolled in the study until the occurrence of the outcomes of interest or until the end of the study period (whichever came first). By applying additional inclusion criteria, T2DM-incident patients were identified as a subgroup of all T2DM-prevalent patients. These patients had at least one outpatient/inpatient T2DM diagnosis recorded in 01/07/2010–31/12/2011 without any previous T2DM diagnosis and without any prescriptions of an antidiabetic agent (ATC groups: A10*) in the preceding 6 months.

### SU monotherapy versus MET monotherapy

**Fig. 1:**
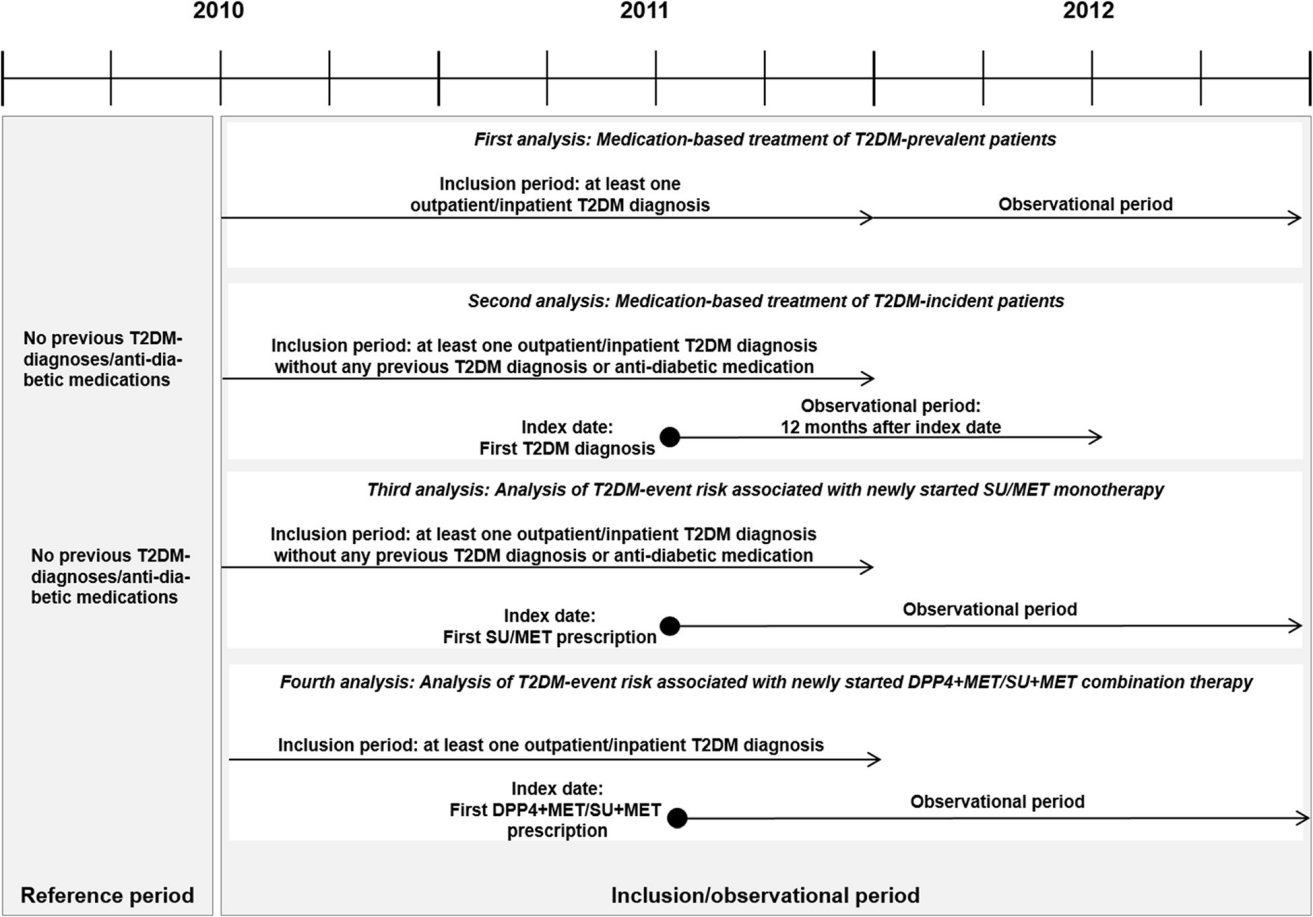
Patient inclusion/exclusion criteria and observational periods for analysed T2DM cohorts

**Fig. 2:**
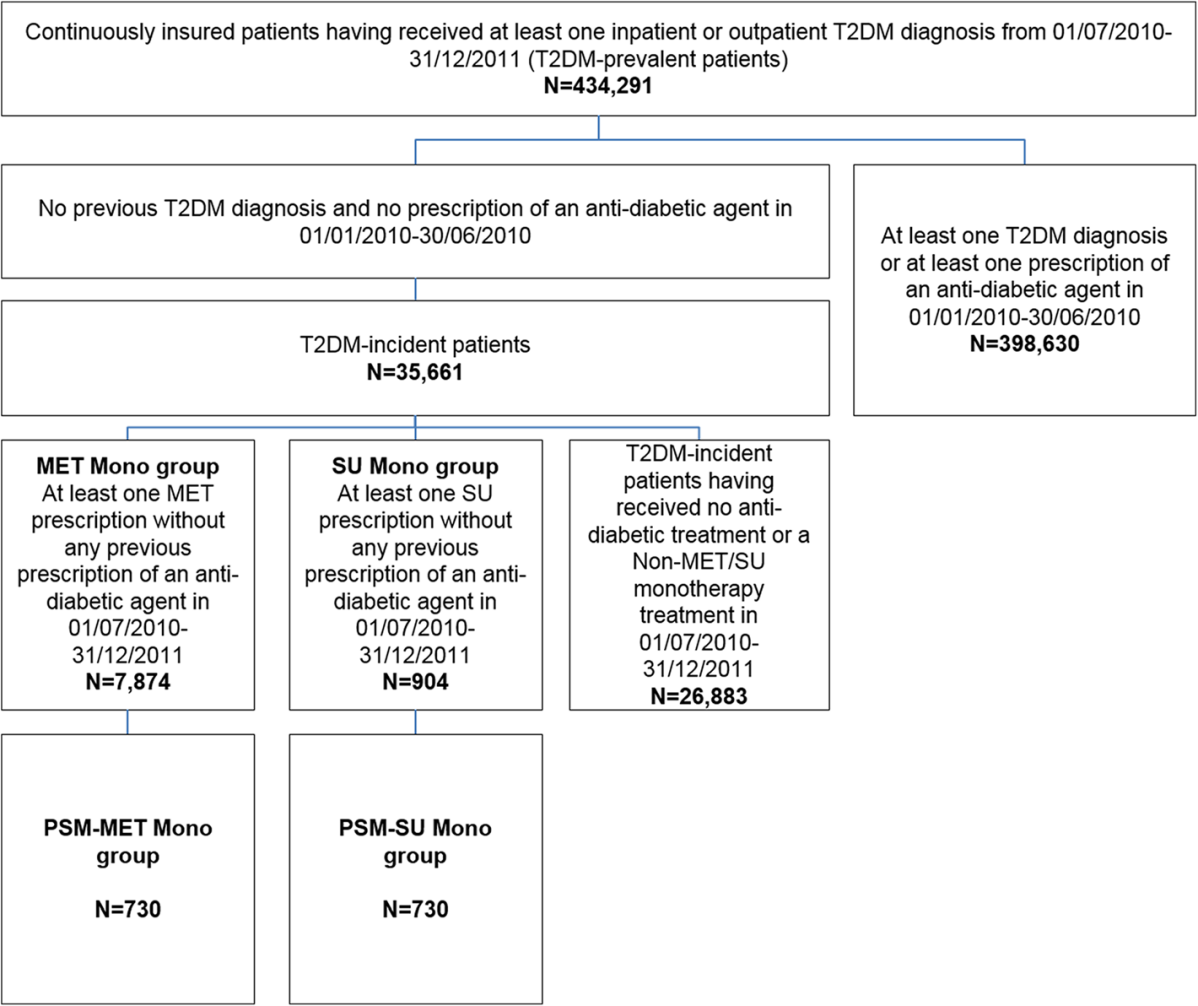
Patient sample of T2DM-incident patients who started SU/MET monotherapy

The study included T2DM-incident patients who started either MET or SU monotherapy between 01/07/2010 and 31/12/2011 without having received any prior antidiabetic medication during the preceding 180 days (Figs. 1 and 2). Observation started with the date of the first observed MET/SU prescription; follow-up time for each patient was at least 12 months (with death as an exception) and lasted until the first observed event, death, therapy discontinuation (treatment gap >180 days or prescription of another agent) or the end of 2012, whichever came first. All patients were followed with regard to the following events:

- MACE
  - ○ Hospitalizations with stroke (ICD-10 codes: I60.-/I61.-/I62.-/I63.-/I64.-)
  - ○ Hospitalizations with acute myocardial infarction (ICD-10 codes: 10 I21.-)
  - ○ Hospitalizations with congestive heart failure (CHF) (ICD-10 codes: 10 I50.-)
  - ○ Hospitalizations with coronary revascularizations (OPS 5-361/5-362/5-363)
  - ○ Hospitalizations with percutaneous transluminal vascular interventions and stent implantations (OPS 8-836/8-837/8-84)
  - ○ Hospitalizations with peripheral vascular disease (ICD-10 code: 10 I73.9)
  - ○ Hospitalizations with angina pectoris (ICD-10 codes: 10 I20.-)
- T2DM-related hospitalizations
  - ○ Hospitalizations with T2DM/acute hypoglycaemia as main diagnosis (ICD-10 codes: E11.-/ E16.0/E16.1/E16.2)
- Death (any cause)
- Composite outcome consisting of MACE, T2DM-related hospitalizations, and all-cause death.

In order to reliably differentiate between acute events and treatment for previous diagnoses/events, this analysis only considered ICD-10 diagnoses or documented procedures (i.e. documented by means of German OPS codes) to represent an event if they were the main motivation for acute hospitalization. The main outcome used in this study was a composite outcome (occurrence of any of the above events); in secondary analyses, the three event types were analysed separately.

### SU+MET combination therapy versus DPP4+MET combination therapy

**Fig. 3:**
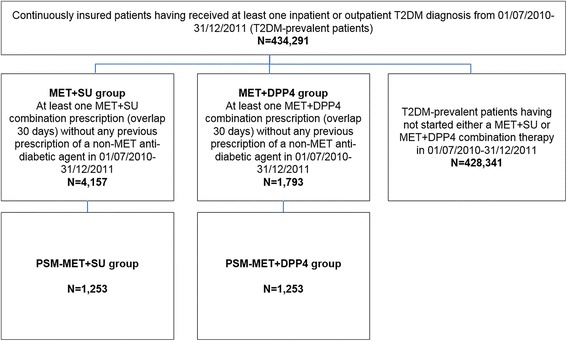
Patient sample of T2DM-prevalent patients who started MET+SU/MET+DPP4 combination therapy

Our analyses of SU+MET combination therapy versus DPP4+MET combination therapy exclusively included T2DM-prevalent patients who had been prescribed MET monotherapy before and who started either MET-SU or MET-DPP4 combination therapy (combination therapy starters; first prescriptions needed to overlap within 30 days) between 01/07/2010 and 31/12/2011 without having received any prior SU/DPP4 medication (for the preceding 180 days). Data are presented in Figs. 1 and 3. Follow-up started with the first observed prescription of the second dual combination agent. All patients were followed with respect to the events as defined above. The follow-up period ended at therapy discontinuation (treatment gap >180 days or prescription of another agent), at death/first observed event or at the end of data availability (31/12/2012).

### Statistical analysis

Differences in event risk for patients who received MET/SU monotherapy or SU+MET/DPP4+MET combination therapy were reported as unadjusted hazard ratios (HRs) in a Cox regression model censoring for death in the analyses addressing time to first MACE and time to first T2DM-related hospitalization and, additionally, censoring for therapy discontinuation/end of follow-up period for all outcome categories including death. Furthermore, the percentage of event-free patients over time was depicted by means of Kaplan Meier (KM) curves, and log-rank tests were used for testing statistical significance of differences.

To address the issue of confounding, two additional analyses were conducted: an analysis of event rates in propensity score matched patient samples and a multivariate Cox regression analysis using time to event as the dependent variable and reporting adjusted HRs (aHRs).

In the propensity score matching (PSM) procedure, SU-exposed patients (either mono or in combination with MET) were matched to SU non-exposed patients (MET mono or DPP4+MET) by propensity score. Only patients with complete (non-imputed) data were included in the analyses. Propensity scores were calculated using logistic regression estimation (with group affiliation as the dependent variable) including age, gender, age-adjusted Charlson Comorbidity Index (CCI; Additional file 1: Table S2) and adapted Diabetes Complications Severity Index (aDCSI; Additional file 2: Table S1) [4] as general independent variables, even if a certain overlap existed between some of these variables. Furthermore, the following variables related to the six months prior to the index prescription were included as independent variables in case these variables significantly influenced group exposition: the number of general practitioner visits, any previous observed micro-/macrovascular complications and prescription of antithrombotic, antihypertensive or lipid lowering medication. A backward elimination approach was used to eliminate any variables that did not reach significance in explaining group exposition; in such cases, these variables were excluded from the PSM model. In any case, models included age, gender and age-adjusted CCI. For the PSM matched cohorts, separate estimates of HRs were calculated following the methodology as described above.

In order to analyse independent factors associated with the observed event risk, additional multivariable Cox regression analyses were conducted covering MET/SU monotherapy patients (Model 1) and SU+MET/DPP4+MET combination therapy patients (Model 2); results were reported as aHRs. In addition to the exposure to either MET/SU monotherapy or SU+MET/DPP4+MET combination therapy, age (as dichotomous variable with a cut-off point at 65 years), gender, age-adjusted CCI and aDCSI were included in these models as independent variables.

All reported *p*-values were two-sided, and 95 % CIs were calculated for HRs/aHRs. All descriptive analyses were performed with Microsoft SQL Server 2008 and Microsoft Excel 2010. All other statistical analyses were performed with SPSS 17.0.

## Results

### T2DM patient characteristics

In our study population, a total of 434,291 T2DM-prevalent and a subgroup of 35,661 T2DM-incident patients were identified (Table 1, Figs. 2 and 3). Of the T2DM-prevalent patients, 56.2 % were female and their mean age was 70.2 years. We also observed a high number of comorbidities per patient in this sample, expressed as a mean CCI (without age factor) of 3.7, which indicates a significant burden in terms of comorbidities experienced per patient.

**Table 1:** Sociodemographic characteristics of observed type 2 diabetes mellitus samples

	*Cohort of T2DM-incident patients*	*T2DM-incident patients who initiated either MET or SU monotherapy*					*Cohort of T2DM-prevalent patients*	*T2DM-prevalent patients who initiated either MET+SU or MET+DPP4 combination therapy*				
		*Unmatched*			*PS-matched*			*Unmatched*			*PS-matched*	
		*SU*	*MET*		*SU*	*MET*		*MET+SU*	*MET+ DPP-4*		*MET+SU*	*MET+ DPP-4*
*N*	*35,661*	*904*	*7,874*		*730*	*730*	*434,291*	*4,157*	*1,793*		*1,253*	*1,253*
*Age in years*	*65.91*	*70.15*	*61.43*	*(p <0.001)*	*67.66*	*67.47*	*70.24*	*68.09*	*62.2*	*(p <0.001)*	*64.61*	*64.8*
*Gender (female)*	*54.17 %*	*54.65 %*	*50.34 %*	*(p <0.050)*	*53.29 %*	*52.47 %*	*56.23 %*	*53.36 %*	*50.81 %*	*(p <0.100)*	*52.35 %*	*51.80 %*
*CCI without age factor (baseline)*	*1.41*	*2.23*	*1.44*	*(p <0.001)*	*1.72*	*1.55*	*3.73*	*2.79*	*2.56*	*(p <0.001)*	*2.41*	*2.47*
*Any macrovascular complications (baseline)*	*1.92 %*	*5.20 %*	*4.19 %*	*(p >0.100)*	*4.79 %*	*4.38 %*	*5.18 %*	*2.09 %*	*3.18 %*	*(p <0.050)*	*1.52 %*	*1.92 %*
*Antithrombotic agent (baseline)*	*15.70 %*	*21.68 %*	*15.76 %*	*(p <0.001)*	*16.58 %*	*15.07 %*	*27.74 %*	*20.88 %*	*18.24 %*	*(p <0.050)*	*17.32 %*	*18.60 %*
*Antihypertensive (baseline)*	*4.75 %*	*5.09 %*	*5.28 %*	*(p >0.100)*	*4.79 %*	*4.11 %*	*9.19 %*	*8.80 %*	*7.36 %*	*(p <0.100)*	*8.06 %*	*7.58 %*
*Lipid lowering drugs (baseline)*	*18.20 %*	*22.68 %*	*22.87 %*	*(p >0.100)*	*22.19 %*	*22.47 %*	*32.94 %*	*32.02 %*	*33.80 %*	*(p >0.100)*	*28.49 %*	*32.00 %*

*Legend:* The table lists the sociodemographic characteristics of the observed samples. These data refer to the start of data availability (01/01/2010) for age/gender and to the 6-month baseline period before the start of observation (for the cohort of T2DM-prevalent patients, this is 01/01/2012)

### SU monotherapy versus MET monotherapy

Of the T2DM-incident patients in our study, 904 patients who were new initiators of SU monotherapy were significantly older (mean age of 70.1 years), were more likely to be female (54.6 %) and had a significantly higher mean age-adjusted CCI (2.23) than the 7,874 therapy-naïve users of MET monotherapy [mean age of 61.4 years (*p* <0.001), 50.3 % female (*p* <0.050), mean age-adjusted CCI of 1.44 (*p* <0.001); Table 1]. We observed 933 patient years of SU monotherapy exposure (mean follow-up period 376.9 days) and 7,850 patient years of MET monotherapy exposure (mean follow-up period 363.9 days).

In the unmatched patient sample comparisons (Table 2; supplemental KM curves in Additional file 3: Figure S3), the HRs (95 % CIs) associated with SU exposure in comparison to MET exposure were 3.3 (2.6–4.3) for mortality, 1.9 (1.4–2.4) for MACE, 3.0 (1.9–4.6) for T2DM-related hospitalizations and 2.5 (2.1–3.0) for composite event risk.

**Table 2:** Crude Hazard Ratios, Hazard Ratios in PSM-matched cohorts and adjusted Hazard Ratios for death, first MACE, first T2DM-related hospitalization and composite outcome in patients treated with SU monotherapy (*n* = 904) versus MET monotherapy (*n* = 7,874); PSM: *n* = 1,253 per group

Events	Crude HR (95 %-CI)	*p*	PSM HR (95 %-CI)	*p*	aHR (95 %-CI)	*p*
Death	3.3 (2.567–4.344)	*<0.001*	1.4 (0.907–2.332)	*0.120*	2.0 (1.538–2.635)	*<0.001*
MACE	1.9 (1.436–2.399)	*<0.001*	1.4 (0.899–2.185)	*0.137*	1.3 (1.033–1.743)	*<0.050*
T2DM-related hospitalization	3.0 (1.927–4.556)	*<0.001*	4.1 (1.551–10.930)	*<0.005*	2.8 (1.807–4.407)	*<0.001*
Composite outcome (any event, whatever came first)	2.5 (2.098–2.995)	*<0.001*	1.6 (1.183–2.259)	*<0.005*	1.8 (1.480–2.132)	*<0.001*

HRs/aHRs reported for SU exposure in comparison to MET exposure

*HR* hazard ratio, *aHR* adjusted hazard ratio, *MET* metformin, *SU* sulphonylureas, *DPP4* dipeptidyl peptidase-4 inhibitor, *PSM* propensity score matching, *CI* confidence interval 95 %

In the PSM comparison which included 1,460 patients (730 patients per group, overlap of propensity scores in Cohort 1, incorporating patients who received MET/SU monotherapy are described in Additional file 4: Figure S1), the HRs (95 % CIs) associated with SU exposure in comparison to MET exposure were 1.4 (0.9–2.3) for mortality, 1.4 (0.9–2.2) for MACE, 4.1 (1.5–10.9) for T2DM-related hospitalizations and 1.6 (1.2–2.3) for composite event rates (Table 2; KM curves in Additional file 3: Fig. S3).

In the multivariable Cox regression models (Table 2; Additional file 5: Figure S5), older age, higher age-adjusted CCI and higher aDCSI were associated with increased MACE/death rates. With respect to hospitalization rates, female gender was associated with lower event rates, while a higher aDCSI was associated with higher event rates. SU monotherapy was associated with higher mortality rates (aHR 2.0; 1.5–2.6), higher MACE rates (aHR 1.3; 1.0–1.7) and higher T2DM-related hospitalization rates (aHR 2.8; 95 % CI: 1.8–4.4). This corresponded with higher composite event rates (aHR 1.8; 1.5–2.1).

### SU+MET combination therapy versus DPP4+MET combination therapy

Among the T2DM-prevalent patients, 4,157 patients who were newly prescribed with a SU+MET combination therapy were significantly older (mean age of 68.1 years), were more likely to be female (53.4 %) and had a significantly higher mean age-adjusted CCI (2.79) than the 1,793 patients with newly prescribed DPP4+MET combination therapy [mean age of 62.2 years (*p* <0.001); 50.8 % female (*p* <0.050) and a mean age-adjusted CCI of 2.56 (*p* <0.001); Table 1]. We observed 4,556 patient years of SU+MET exposure (mean follow-up period of 400.0 days) and 1,752 patient years of DPP4+MET exposure (mean follow-up period of 356.6 days).

In the unmatched patient sample comparisons (Table 3; Additional file 6: Figure S4), estimated HRs (95 % CIs) associated with SU+MET exposure in comparison to MET+DPP4 exposure were 1.5 (1.0–2.4) for mortality, 1.0 (0.8–1.4) for MACE, 0.9 (0.6–1.5) for T2DM hospitalizations, and 1.1 (0.9–1.3) for composite event rates.

**Table 3:** Crude Hazard Ratios, Hazard Ratios in PSM-matched cohorts and adjusted Hazard Ratios for death, first MACE, first T2DM-related hospitalization and composite outcome in patients treated with SU + MET (*n* = 4,157) versus DPP-4 + MET (*n* = 1,793); PSM: *n* = 1,253 per group

Events	Crude HR (95 %-CI)	*p*	PSM HR (95 %-CI)	*p*	aHR (95 %-CI)	*p*
Death	1.5 (0.966–2.414)	*0.070*	1.3 (0.662–2.596)	*0.437*	1.3 (0.792–2.005)	*0.330*
MACE	1.0 (0.804–1.362)	*0.736*	0.7 (0.487–1.123)	*0.157*	0.8 (0.650–1.110)	*0.850*
T2DM-related hospitalization	0.9 (0.588–1.446)	*0.725*	0.9 (0.446–1.679)	*0.668*	0.8 (0.527–1.320)	*0.438*
Composite outcome (any event, whatever came first)	1.1 (0.883–1.344)	*0.425*	0.8 (0.616–1.167)	*0.313*	0.9 (0.734–1.126)	*0.382*

HRs/aHRs reported for SU+MET exposure in comparison to DPP4+MET exposure

*HR* hazard ratio, *aHR* adjusted hazard ratio, *MET* metformin, *SU* sulphonylureas, *DPP4* dipeptidyl peptidase-4 inhibitor, *PSM* propensity score matching, *CI* confidence interval 95 %

In the PSM comparison which included 2,506 patients (1,253 patients per group, overlap of propensity scores in Cohort 2, incorporating patients who received SU+MET and DPP4-MET combination therapy are described in Additional file 7: Figure S2), HRs (95 % CIs) associated with SU+MET exposure were 1.3 (0.7–2.6) for mortality, 0.7 (0.5–1.1) for MACE, 0.9 (0.4–1.7) for T2DM hospitalizations and 0.8 (0.6–1.2) for composite event rates (Table 3; Additional file 8: Figure S8). In the multivariable Cox regression models (Table 3; Additional file 9: Figure S6), older age, higher age-adjusted CCI, higher aDSCI and male gender were associated with an increased risk of all-cause events (including MACE, deaths and T2DM-related hospitalizations). When we compared SU+MET combination therapy to DPP4+MET combination therapy, as was done in the PSM analysis, no statistically significant results were found (Table 3; Additional file 10: Figure S7).

## Discussion

The results of this study indicate that SU monotherapy may be associated with an increased risk of death, MACE and hospitalizations for T2DM patients compared to MET monotherapy, taking into account the differences in patient characteristics. This was seen in crude as well as multivariate Cox regression analyses, but due to small sample sizes this could not be confirmed for all observed outcomes in the PSM comparison. However, point estimates indicated similar associations, and we also observed a trend of non-significant increased risk for MACE and death in the SU group in the PSM comparison. Furthermore, there was a significantly higher risk of T2DM-related hospitalizations in the SU group in the PSM analysis which also translated, together with the aforementioned results, into a lower percentage of PS-matched patients treated with MET experiencing an all-cause event.

The higher SU-associated T2DM hospitalization risk may underpin the disadvantage of higher rates of hypoglycaemia associated with SU therapy [20], something which has also been confirmed by earlier studies and reported in a recently published review [21–23]. In addition, another systematic review and meta-analysis as well as another study found that patients receiving SU treatment had an increased all-cause mortality risk [16, 24]; this, however, could not be confirmed in every study [25]. Furthermore, a UK-based study which was very similar to the one reported here compared MACE/mortality risk among T2DM-incident patients treated with either SU or MET monotherapy; this study did not include T2DM-related hospitalizations as an event type. It concluded that SU monotherapy was associated with increased MACE/mortality risk [11]. Another study which examined SU monotherapy in T2DM patients in comparison to MET monotherapy reported that SU users experienced treatment failure (defined as progression to a combination of oral anti-hyperglycaemia drug therapy, insulin use or an HbA1C >7.5 %) significantly earlier and more frequently than MET monotherapy users [26].

While further examinations of potential risk factors related to an increased mortality/MACE/hospitalization risk associated with SU monotherapy are not available in this current study, evidence from previous studies indicates that several factors may contribute to the underlying risks, including weight gain [27–30], links to cancer [31, 32], increased insulin resistance and the underliying SU mechanism of action [33–36].

A German analysis covering data provided by 1,201 GPs reported a lower macrovascular event frequency under DPP4 treatment in comparison to SU treatment. This could not be confirmed in our study. However, in this study, events were identified through GP diagnoses only; these may have described more existing co-morbidities in T2DM patients than incident events in our definition, which identified events through acute hospitalizations only. Moreover, we observed patients who received either SU or MET monotherapy or SU+MET or DPP4+MET combination therapy only, whereas this analysis only excluded concomitant insulin therapy but allowed for all other antidiabetic agents [24]. Furthermore, our analysis covered prescriptions and outpatient treatment by a larger number of physicians (12,419 outpatient physicians, with 5,055 different GPs and outpatient specialists involved).

In contrast to our study, a similar analysis based on a retrospective sample of UK patients found all-cause mortality to be lower in the DPP4+MET group; a similar trend was also observed for MACE risk [24]. This UK analysis was based on a significantly larger sample size of 33,983 MET+SU and 7,864 MET+DPP4 patients in the unmatched comparison and 13,802 patients in the PSM comparison. In addition, median follow-up time was also longer in the UK study when compared to our study. Furthermore, the patient characteristics in our study also differed significantly from the UK analysis: whereas mean age in our PSM cohorts was 64.6–64.8 years, mean age in the UK-PSM cohorts was 59.8–60.4 years. Results similar to the abovementioned UK analysis were found in another large study [24]; our results were confirmed in several other retrospective database studies [24, 37].

There may be specific clinical reasons why SU/MET+SU patients received this specific type of therapy (e.g. low risk of hypoglycaemia). In choosing SU therapy, MET contraindications may have played a major role. We observed MET contraindications in 44 % of the patients contained in our database. This may also explain event/mortality rate differences in the unmatched comparisons between MET/SU and MET+SU/MET+DPP4 groups. Other reasons for choosing a specific antidiabetic therapy were unknown to us, but could have confounded the results. Furthermore, we observed comparatively old/comorbid T2DM patients. This is due to the characteristics of those insured in the health care fund which provided the data. This means that T2DM patients with higher comorbidity levels are over-represented in our study.

Our data show that patients receiving SU therapy (mono or combo) differ significantly from other T2DM patients treated with MET in any combination: they tend to be older, have greater comorbidity and are more often female. So, for example, the mean ages of patients who received MET mono, GLP-1+OAD, GLP-1+OAD+insulin or MET+DPP4 combination therapy were 69.0, 57.5, 58.0 and 66.8 years, respectively. In comparison, the mean ages of patients who received SU mono or SU+MET combination therapy were 76.8 and 72.2 years, respectively. This makes a real-world comparison of SU with GLP-1s/DPP4s a challenging task because, obviously, new antidiabetic agents address completely different T2DM patient cohorts in real-life practice than SUs. Consequently, a substantial number of patients were excluded in the PSM comparisons. To reduce the bias risk for those patients included in the PSM cohorts, we used all available variables that significantly influenced group exposition, even if there was a certain overlap between these variables, as was the case with CCI and aDCSI.

### Limitations

The current study is an observational cohort study with several limitations commonly associated with observational studies. First of all, it is limited with regard to its sample size and, more importantly, to the relatively short duration of follow-up. In addition, a significant number of patients was lost to follow-up, with 42.8 % of SU monotherapy patients and 47.8 % of SU+MET combination therapy patients discontinuing their treatment (treatment gap >180 days or prescription of another agent) or suffering a fatal event within the first 12 months after initiation of therapy. Thus, these patients had a much shorter follow-up than the planned minimum period of 12 months, and they were censored prematurely before the end of the study period.

Finally, certain information concerning several risk factors known from patient demographics and clinical characteristics associated with event risk were not available in our claims data. This included information about HbA1C [9, 38–40] and blood pressure [41, 42], which may predict MACE/mortality based on a U-curve pattern [3]. It also included preclinical atherosclerosis [43], specific GFR values [44], level of physical activity [45] and total or low density lipoprotein (LDL)-cholesterol values, which have been found to be independent cardiovascular risk factors in other T2DM studies [38, 46].

## Conclusions

Our study suggests that SU monotherapy may be associated with an increased risk of mortality, MACE, T2DM hospitalizations and/or all-cause events, compared to MET monotherapy. Current German and European guidelines mostly recommend the use of SU as second-line therapy or, in case of MET contraindications, the use of SU as first-line therapy, and SU therapy is still prescribed in an important part of T2DM patients in Germany [8, 9]. Our results indicate that in considering SU therapy, the associated cardiovascular risk should also be taken into account.

## Abbreviations

AD medication, antidiabetic medication; aDCSI, adapted diabetes complications severity index; aHR, adjusted Hazard ratio; ATC, anatomical therapeutic chemical; CCI, Charlson comorbidity index; DMP, disease management programme; DPP-4, dipeptidyl peptidase-4; GLP-1, glucagon-like peptide-1; HR, Hazard ratio; ICD, International statistical classification of diseases; IRR, incidence rate ratio; KM, Kaplan Meier; MACE, macrovascular event; MET, metformin; OAD, oral antidiabetic drugs; PS, propensity score; SU, sulfonyl urea; T2DM, type 2 diabetes mellitus

## Additional files

Additional file 1: Table S1. Components of the aDSCI. The table contains the components of the adapted Diabetes Complications Severity Index and describes the score methodology used, based on observed outpatient/inpatient ICD-10 codes in 2010. (TIF 141 kb)

Additional file 2: Table S2. Charlson Comorbidity Index (CCI) and its components. The table outlines the components of the Charlson Comorbidity Index (CCI) and describes the score methodology used, based on observed outpatient/inpatient ICD-10 codes in 2010. (TIF 122 kb)

Additional file 3: Figure S3. Kaplan-Meier (KM) curves for crude all-cause death rates, macrovascular event rates and T2DM-related hospitalizations for patients with either MET or SU monotherapy. The figure shows KM curves representing the percentage of event-free patients (all-cause event as well as mortality, MACE and T2DM-related hospitalizations) for two T2DM-incident cohorts: patients who received SU monotherapy and patients who received MET monotherapy. Observation started with the first observed SU/MET prescription. (TIF 498 kb)

Additional file 4: Figure S1. Distribution of propensity scores as calculated by logistic regression for MET/SU monotherapy users. This figure describes the overlap of propensity scores in Cohort 1, incorporating patients who received MET/SU monotherapy. (TIF 198 kb)

Additional file 5: Figure S5. Multivariable Cox regression models estimating time to event for four outcome categories (MET/SU monotherapy). The figure shows the results of the multivariable Cox regression analysis with regard to independent factors influencing time until an event (all-cause event as well as mortality, MACE and T2DM-related hospitalizations in separate models) in the T2DM-incident sample that received either SU or MET monotherapy. (TIF 164 kb)

Additional file 6: Figure S4. Kaplan-Meier (KM) curves for all-cause death rates, macrovascular event rates and T2DM-related hospitalizations for patients with either MET or SU monotherapy (PS matched groups). The figure shows KM curves representing the percentage of event-free patients (all-cause event as well as mortality, MACE and T2DM-related hospitalizations) for two T2DM-incident cohorts: patients who received SU monotherapy and patients who received MET monotherapy. Cohorts are matched by PSM. Observation started with the first observed SU/MET prescription. (TIF 546 kb)

Additional file 7: Figure S2. Distribution of propensity scores as calculated by logistic regression for SU+MET and DPP4-MET combination therapy users. This figure describes the overlap of propensity scores in Cohort 2, incorporating patients who received SU+MET or DPP4-MET combination therapy. (TIF 227 kb)

Additional file 8: Figure S8. Multivariable Cox regression models estimating time to event for four outcome categories (MET+SU/MET+DPP-4 therapy). Factors associated with event risk. The figure shows the results of the multivariable Cox regression analysis with regard to independent factors influencing time until an event (all-cause event as well as mortality, MACE and T2DM-related hospitalizations in separate models) in the T2DM-prevalent sample that received either SU+MET or DPP4+MET combination therapy. (TIF 160 kb)

Additional file 9: Figure S6. Kaplan-Meier (KM) curves for crude all-cause death rates, macrovascular event rates and T2DM-related hospitalizations for patients with either MET+SU or MET+DPP-4 therapy. The figure shows KM curves representing the percentage of event-free patients (all-cause event as well as mortality, MACE and T2DM-related hospitalizations) for the two cohorts defined above. Observation started with the first observed prescription of the second combination agent. (TIF 604 kb)

Additional file 10: Figure S7. Kaplan-Meier (KM) curves for crude all-cause death rates, macrovascular event rates and T2DM-related hospitalizations for patients with either MET+SU or MET+DPP-4 therapy (PS matched groups). The figure shows KM curves representing the percentage of event-free patients (all-cause event as well as mortality, MACE and T2DM-related hospitalizations) for the two cohorts defined above. Observation started with the first observed prescription of the second combination agent. (TIF 629 kb)

## References

[1] Robert Koch-Institut (RKI). Daten und Fakten: Ergebnisse der Studie “Gesundheit in Deutschland aktuell 2009” - Beiträge zur GBE [cited 2016 Mar 4]. Available from: URL:https://www.rki.de/DE/Content/Gesundheitsmonitoring/Gesundheitsberichterstattung/GBEDownloadsB/GEDA09.pdf?__blob=publicationFile.

[2] Wilke T, Groth A, Fuchs A, Seitz L, Kienhöfer J, Lundershausen R (2014). Real life treatment of diabetes mellitus type 2 patients: an analysis based on a large sample of 394,828 German patients. Diabetes Res Clin Pract.

[3] Wilke T, Mueller S, Groth A, Fuchs A, Seitz L, Kienhöfer J (2015). Treatment-dependent and treatment-independent risk factors associated with the risk of diabetes-related events: a retrospective analysis based on 229,042 patients with type 2 diabetes mellitus. Cardiovasc Diabetol.

[4] Chang HY, Weiner JP, Richards TM, Bleich SN, Segal JB (2012). Validating the adapted Diabetes Complications Severity Index in claims data. Am J Manag Care.

[5] Young BA, Lin E, von Korff M, Simon G, Ciechanowski P, Ludman EJ (2008). Diabetes complications severity index and risk of mortality, hospitalization, and healthcare utilization. Am J Manag Care.

[6] Norgaard ML, Andersen SS, Schramm TK, Folke F, Jørgensen CH, Hansen ML (2010). Changes in short- and long-term cardiovascular risk of incident diabetes and incident myocardial infarction—a nationwide study. Diabetologia.

[7] Liebl A, Neiss A, Spannheimer A, Reitberger U, Wagner T, Gortz A (2001). Costs of type 2 diabetes in Germany. Results of the CODE-2 study. Dtsch Med Wochenschr.

[8] Bundesärztekammer (BÄK), Kassenärztliche Bundesvereinigung (KBV), Arbeitsgemeinschaft der Wissenschaftlichen Medizinischen Fachgesellschaften (AWMF). Nationale VersorgungsLeitlinie Therapie des Typ-2-Diabetes. Langfassung [cited 2016 Mar 4]. Available from: URL:http://www.deutsche-diabetes-gesellschaft.de/fileadmin/Redakteur/Leitlinien/Evidenzbasierte_Leitlinien/NVL_Typ-2_Therapie-lang_Apr_2014.pdf.

[9] International Diabetes Federation Guideline Development Group (2014). Global guideline for type 2 diabetes. Diabetes Res Clin Pract.

[10] Desai NR, Shrank WH, Fischer MA, Avorn J, Liberman JN, Schneeweiss S (2012). Patterns of medication initiation in newly diagnosed diabetes mellitus: quality and cost implications. Am J Med.

[11] Morgan CL, Mukherjee J, Jenkins-Jones S, Holden SE, Currie CJ (2014). Association between first-line monotherapy with sulphonylurea versus metformin and risk of all-cause mortality and cardiovascular events: a retrospective, observational study. Diabetes Obes Metab.

[12] Gemeinsamer Bundesausschuss. Beschluss des Gemeinsamen Bundesausschusses über eine Änderung der Arzneimittel-Richtlinie (AM-RL): Anlage XII – Beschlüsse über die Nutzenbewertung von Arzneimitteln mit neuen Wirkstoffen nach § 35a SGB V – Saxagliptin [cited 2016 Mar 4]. Available from: URL:https://www.g-ba.de/downloads/40-268-2577/2013-05-02_AM-RL-XII_Saxagliptin%20Metformin_ZD.pdf.

[13] Gemeinsamer Bundesausschuss. Beschluss des Gemeinsamen Bundesausschusses über eine Änderung der Arzneimittel-Richtlinie (AM-RL): Anlage XII – Beschlüsse über die Nutzenbewertung von Arzneimitteln mit neuen Wirkstoffen nach § 35a SGB V – Sitagliptin; 2013 [cited 2016 Mar 4]. Available from: URL:https://www.g-ba.de/downloads/40-268-2966/2013-10-01_AM-RL-XII_Sitagliptin_ZD.pdf.

[14] Bundesministerium der Justiz. Bundesministerium für Gesundheit - Bekanntmachung eines Beschlusses des Gemeinsamen Bundesausschusses über eine Änderung der Arzneimittel-Richtlinie (AM-RL): Anlage XII - Beschlüsse über die Nutzenbewertung von Arzneimitteln mit neuen Wirkstoffen nach § 35a des Fünften Buches Sozialgesetzbuch (SGB V) Linagliptin; 2012 [cited 2016 Mar 4]. Available from: URL:https://www.g-ba.de/downloads/40-268-1919/2012-03-29_AM-RL-XII_Linagliptin_ZD.pdf.

[15] Hemmingsen B, Schroll JB, Lund SS, Wetterslev J, Gluud C, Vaag A, Hemmingsen B (1996). Sulphonylurea monotherapy for patients with type 2 diabetes mellitus. Cochrane Database of Systematic Reviews.

[16] Forst T, Hanefeld M, Jacob S, Moeser G, Schwenk G, Pfutzner A (2013). Association of sulphonylurea treatment with all-cause and cardiovascular mortality: a systematic review and meta-analysis of observational studies. Diab Vasc Dis Res.

[17] Garratt KN, Brady PA, Hassinger NL, Grill DE, Terzic A, Holmes JR (1999). Sulfonylurea drugs increase early mortality in patients with diabetes mellitus after direct angioplasty for acute myocardial infarction. J Am Coll Cardiol.

[18] Simpson SH, Majumdar SR, Tsuyuki RT, Eurich DT, Johnson JA (2006). Dose-response relation between sulfonylurea drugs and mortality in type 2 diabetes mellitus: a population-based cohort study. CMAJ.

[19] Morgan CL, Mukherjee J, Jenkins-Jones S, Holden SE, Currie CJ (2014). Combination therapy with metformin plus sulphonylureas versus metformin plus DPP-4 inhibitors: association with major adverse cardiovascular events and all-cause mortality. Diabetes Obes Metab.

[20] Cronin O, Morris WPJ, Golledge J (2013). The association of obesity with cardiovascular events in patients with peripheral artery disease. Atherosclerosis.

[21] Ionova T, Nikitina T, Kurbatova K, Rodionova A (2015). Benefits and risks of Vildagliptin/Metformin versus Sulphonylureas/Metformin combination therapy in Type 2 Diabetes Mellitus (T2DM) from patient’s perspective: real-world data. Value Health.

[22] Barnett AH, Charbonnel B, Moses RG, Kalra S (2015). Dipeptidyl peptidase-4 inhibitors in triple oral therapy regimens in patients with type 2 diabetes mellitus. Curr Med Res Opin.

[23] Rathmann W, Kostev K, Gruenberger JB, Dworak M, Bader G, Giani G (2013). Treatment persistence, hypoglycaemia and clinical outcomes in type 2 diabetes patients with dipeptidyl peptidase-4 inhibitors and sulphonylureas: a primary care database analysis. Diabetes Obes Metab.

[24] Ou S, Shih C, Chao P, Chu H, Kuo S, Lee Y (2015). Effects on clinical outcomes of adding dipeptidyl peptidase-4 inhibitors versus sulfonylureas to metformin therapy in patients with type 2 diabetes mellitus. Ann Intern Med.

[25] Gallwitz B, Thiemann S, Wörle H, Marx N (2015). Kardiovaskuläre Studien-Endpunkte bei Typ-2-Diabetes und die Sulfonylharnstoff-Kontroverse. Dtsch Med Wochenschr.

[26] Jiang G, Luk AO, Yang X, Wang Y, Tam CH, Lau SH (2016). Progression to treatment failure among Chinese patients with type 2 diabetes initiated on metformin versus sulphonylurea monotherapy—The Hong Kong Diabetes Registry. Diabetes Res Clin Pract.

[27] Phung OJ, Scholle JM, Talwar M, Coleman CI (2010). Effect of noninsulin antidiabetic drugs added to metformin therapy on glycemic control, weight gain, and hypoglycemia in type 2 diabetes. JAMA.

[28] Anderson JW, Konz EC (2001). Obesity and disease management: effects of weight loss on comorbid conditions. Obes Res.

[29] Nathan DM, Buse JB, Davidson MB, Ferrannini E, Holman RR, Sherwin R (2008). Medical management of hyperglycemia in type 2 diabetes: a consensus algorithm for the initiation and adjustment of therapy: a consensus statement of the American diabetes association and the European association for the study of diabetes. Diabetes Care.

[30] Turner R, UK Prospective Diabetes Study Group (1998). Tight blood pressure control and risk of macrovascular and microvascular complications in type 2 diabetes: UKPDS 38. UK Prospective Diabetes Study Group. BMJ.

[31] Gallagher EJ, LeRoith D (2010). The proliferating role of insulin and insulin-like growth factors in cancer. Trends Endocrinol Metab.

[32] Currie CJ, Poole CD, Gale EAM (2009). The influence of glucose-lowering therapies on cancer risk in type 2 diabetes. Diabetologia.

[33] Abdella NA (2002). Controversies in management of diabetes in patients with coronary heart disease. Med Princ Pract.

[34] Engler RL, Yellon DM (1996). Sulfonylurea KATP blockade in type II diabetes and preconditioning in cardiovascular disease. Time for reconsideration. Circulation.

[35] Smith SA, Porter L, Biswas N, Freed MI (2004). Rosiglitazone, but not glyburide, reduces circulating proinsulin and the proinsulin:insulin ratio in type 2 diabetes. J Clin Endocrinol Metab.

[36] Cao W, Ning J, Yang X, Liu Z (2011). Excess exposure to insulin is the primary cause of insulin resistance and its associated atherosclerosis. Curr Mol Pharmacol.

[37] Kim SC, Glynn RJ, Liu J, Everett BM, Goldfine AB (2014). Dipeptidyl peptidase-4 inhibitors do not increase the risk of cardiovascular events in type 2 diabetes: a cohort study. Acta Diabetol.

[38] Currie CJ, Peters JR, Tynan A, Evans M, Heine RJ, Bracco OL (2010). Survival as a function of HbA1c in people with type 2 diabetes: a retrospective cohort study. Lancet.

[39] Stone MA, Charpentier G, Doggen K, Kuss O, Lindblad U, Kellner C (2013). Quality of care of people with type 2 diabetes in eight European Countries: findings from the guideline adherence to enhance care (GUIDANCE) study. Diabetes Care.

[40] Müller N, Heller T, Freitag MH, Gerste B, Haupt CM, Wolf G (2015). Healthcare utilization of people with type 2 diabetes in Germany: an analysis based on health insurance data. Diabet Med.

[41] Zoungas S, de Galan BE, Ninomiya T, Grobbee D, Hamet P, Heller S (2009). Combined effects of routine blood pressure lowering and intensive glucose control on macrovascular and microvascular outcomes in patients with type 2 diabetes: new results from the ADVANCE trial. Diabetes Care.

[42] Hata J, Arima H, Rothwell PM, Woodward M, Zoungas S, Anderson C (2013). Effects of visit-to-visit variability in systolic blood pressure on macrovascular and microvascular complications in patients with type 2 diabetes mellitus: the ADVANCE trial. Circulation.

[43] Novo S, Peritore A, Trovato R, Guarneri F, Di Lisi D, Muratori I (2013). Preclinical atherosclerosis and metabolic syndrome increase cardio- and cerebrovascular events rate: a 20-year follow up. Cardiovasc Diabetol.

[44] Fabbian F, de Giorgi A, Monesi M, Pala M, Tiseo R, Misurati E (2014). All-cause mortality and estimated renal function in type 2 diabetes mellitus outpatients: is there a relationship with the equation used?. Diab Vasc Dis Res.

[45] Zethelius B, Gudbjornsdottir S, Eliasson B, Eeg-Olofsson K, Cederholm J (2014). Level of physical activity associated with risk of cardiovascular diseases and mortality in patients with type-2 diabetes: report from the Swedish National Diabetes Register. Eur J Prev Cardiol.

[46] Fruchart J, Davignon J, Hermans MP, Al-Rubeaan K, Amarenco P, Assmann G (2014). Residual macrovascular risk in 2013: what have we learned?. Cardiovasc Diabetol.

