# Supplementary figures and images for: Effectiveness of sulphonylureas in the therapy of diabetes mellitus type 2 patients: an observational cohort study
Source: J Diabetes Metab Disord. 2016 Aug 2;15:28. doi: 10.1186/s40200-016-0251-9 (PMC4969981; doi:10.1186/s40200-016-0251-9)

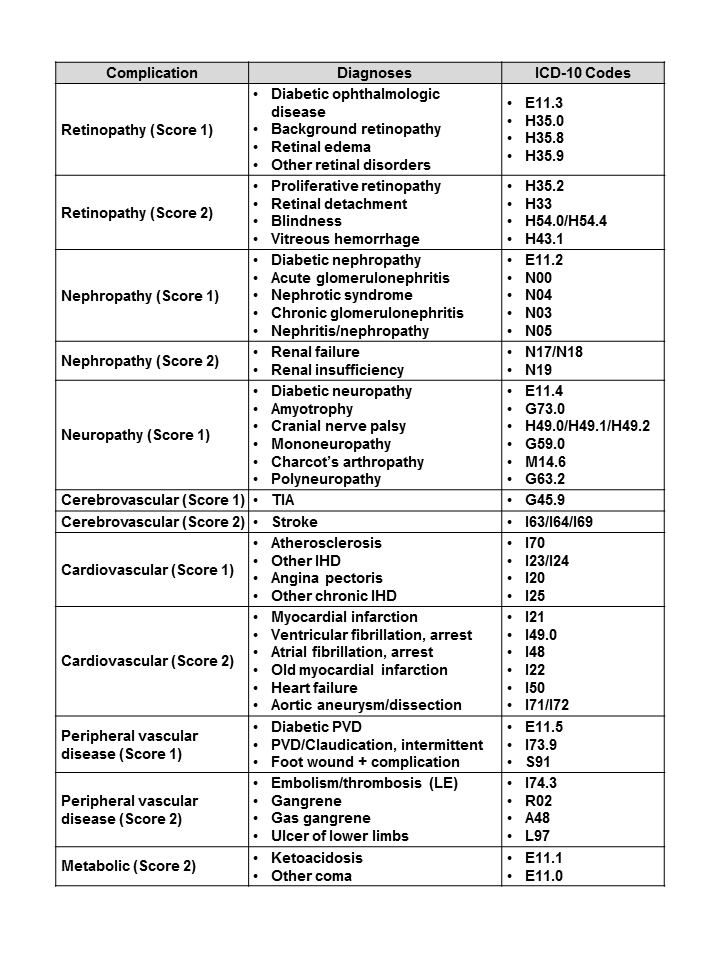

Supplement: Supplementary file 1 — Components of the aDSCI. The table contains the components of the adapted Diabetes Complications Severity Index and describes the score methodology used, based on observed outpatient/inpatient ICD-10 codes in 2010. (TIF 141 kb) [file 40200_2016_251_MOESM1_ESM.tif]

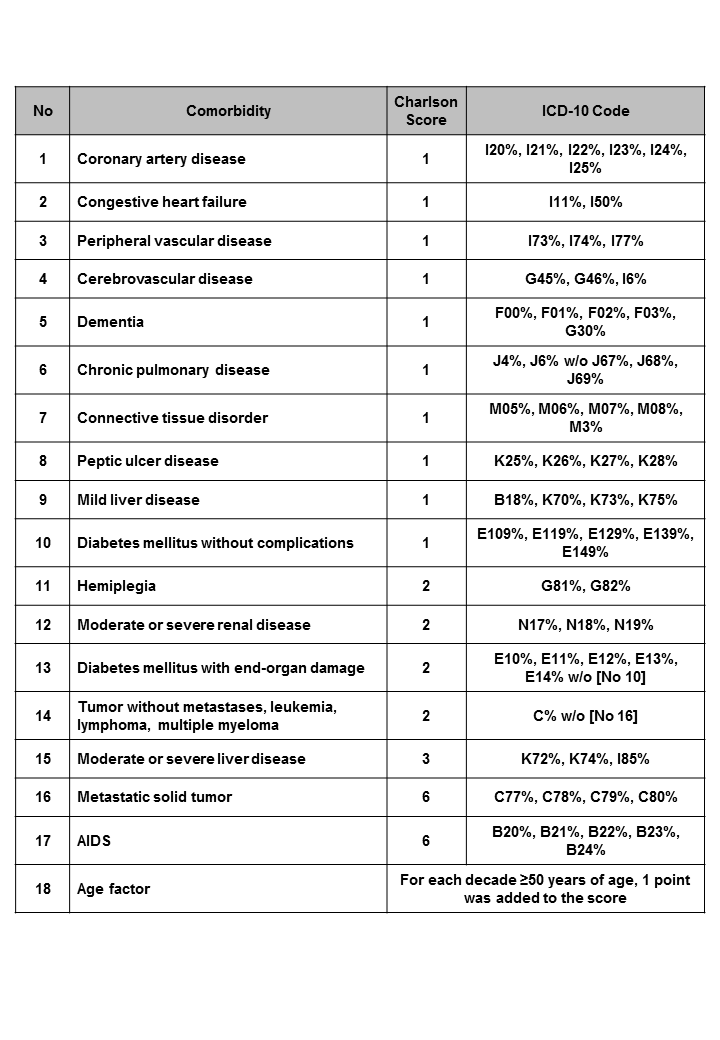

Supplement: Supplementary file 2 — Charlson Comorbidity Index (CCI) and its components. The table outlines the components of the Charlson Comorbidity Index (CCI) and describes the score methodology used, based on observed outpatient/inpatient ICD-10 codes in 2010. (TIF 122 kb) [file 40200_2016_251_MOESM2_ESM.tif]

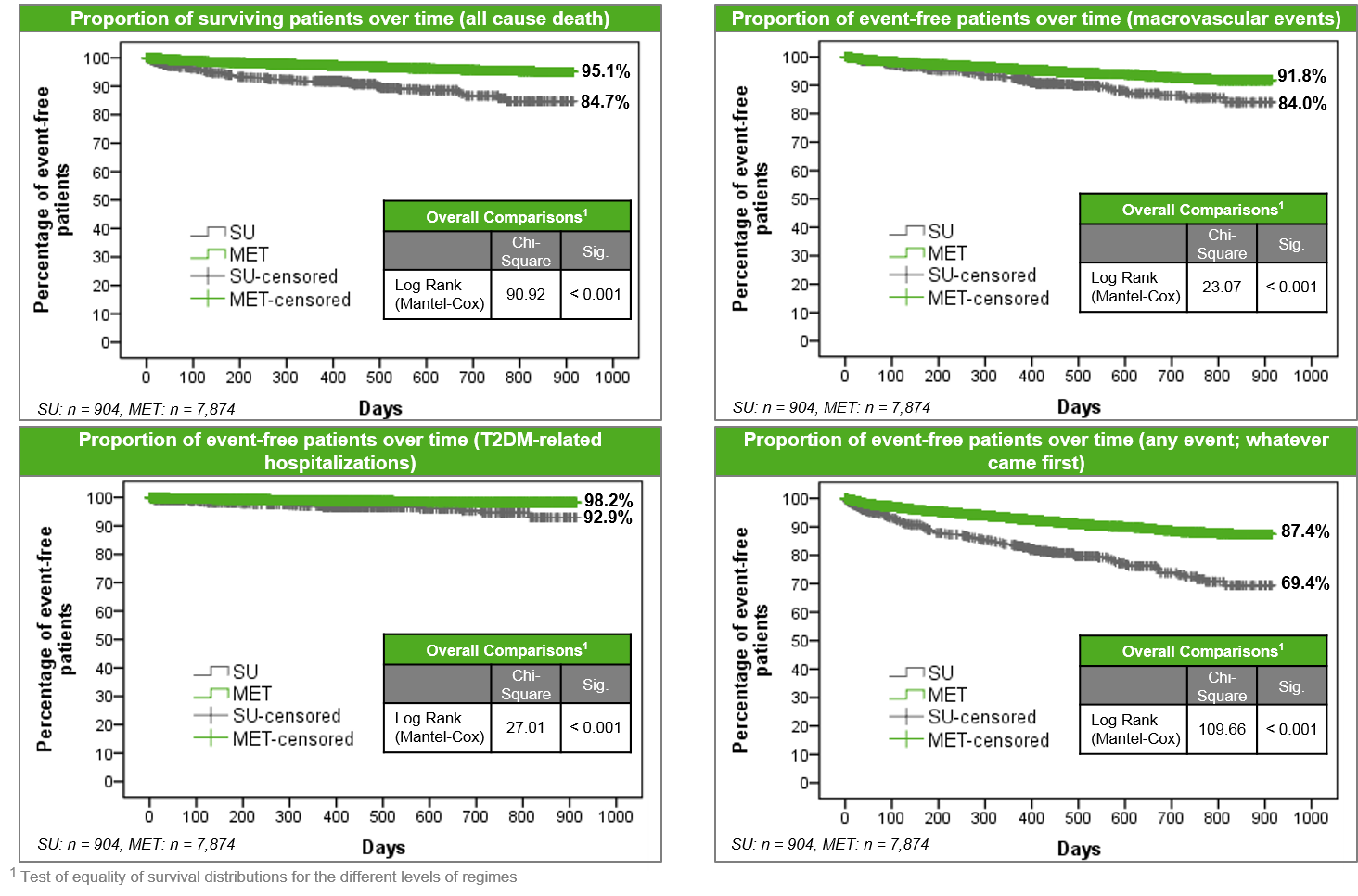

Supplement: Supplementary file 3 — Kaplan-Meier (KM) curves for crude all-cause death rates, macrovascular event rates and T2DM-related hospitalizations for patients with either MET or SU monotherapy. The figure shows KM curves representing the percentage of event-free patients (all-cause event as well as mortality, MACE and T2DM-related hospitalizations) for two T2DM-incident cohorts: patients who received SU monotherapy and patients who received MET monotherapy. Observation started with the first observed SU/MET prescription. (TIF 498 kb) [file 40200_2016_251_MOESM3_ESM.tif]

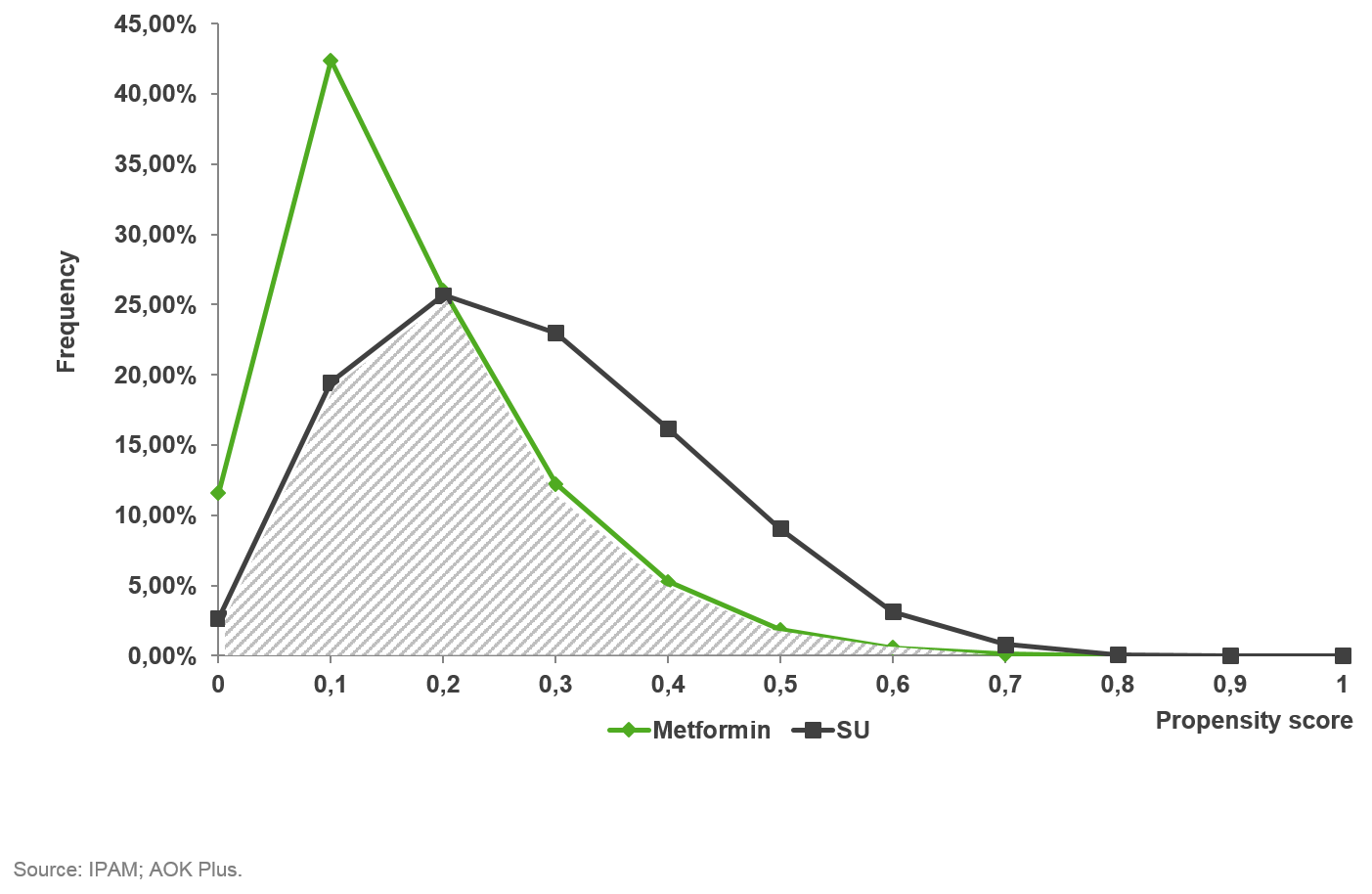

Supplement: Supplementary file 4 — Distribution of propensity scores as calculated by logistic regression for MET/SU monotherapy users. This figure describes the overlap of propensity scores in Cohort 1, incorporating patients who received MET/SU monotherapy. (TIF 198 kb) [file 40200_2016_251_MOESM4_ESM.tif]

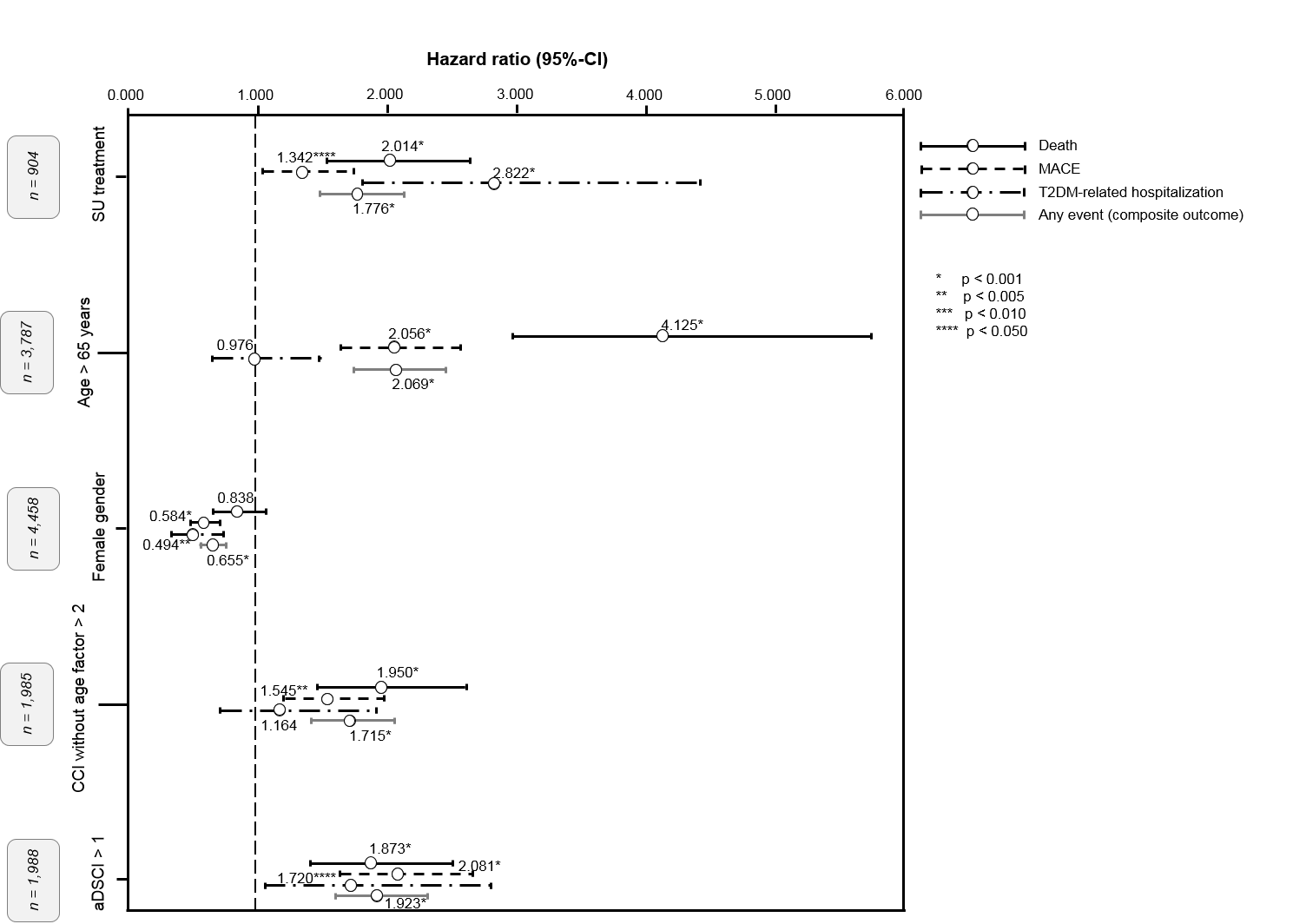

Supplement: Supplementary file 5 — Multivariable Cox regression models estimating time to event for four outcome categories (MET/SU monotherapy). The figure shows the results of the multivariable Cox regression analysis with regard to independent factors influencing time until an event (all-cause event as well as mortality, MACE and T2DM-related hospitalizations in separate models) in the T2DM-incident sample that received either SU or MET monotherapy. (TIF 164 kb) [file 40200_2016_251_MOESM5_ESM.tif]

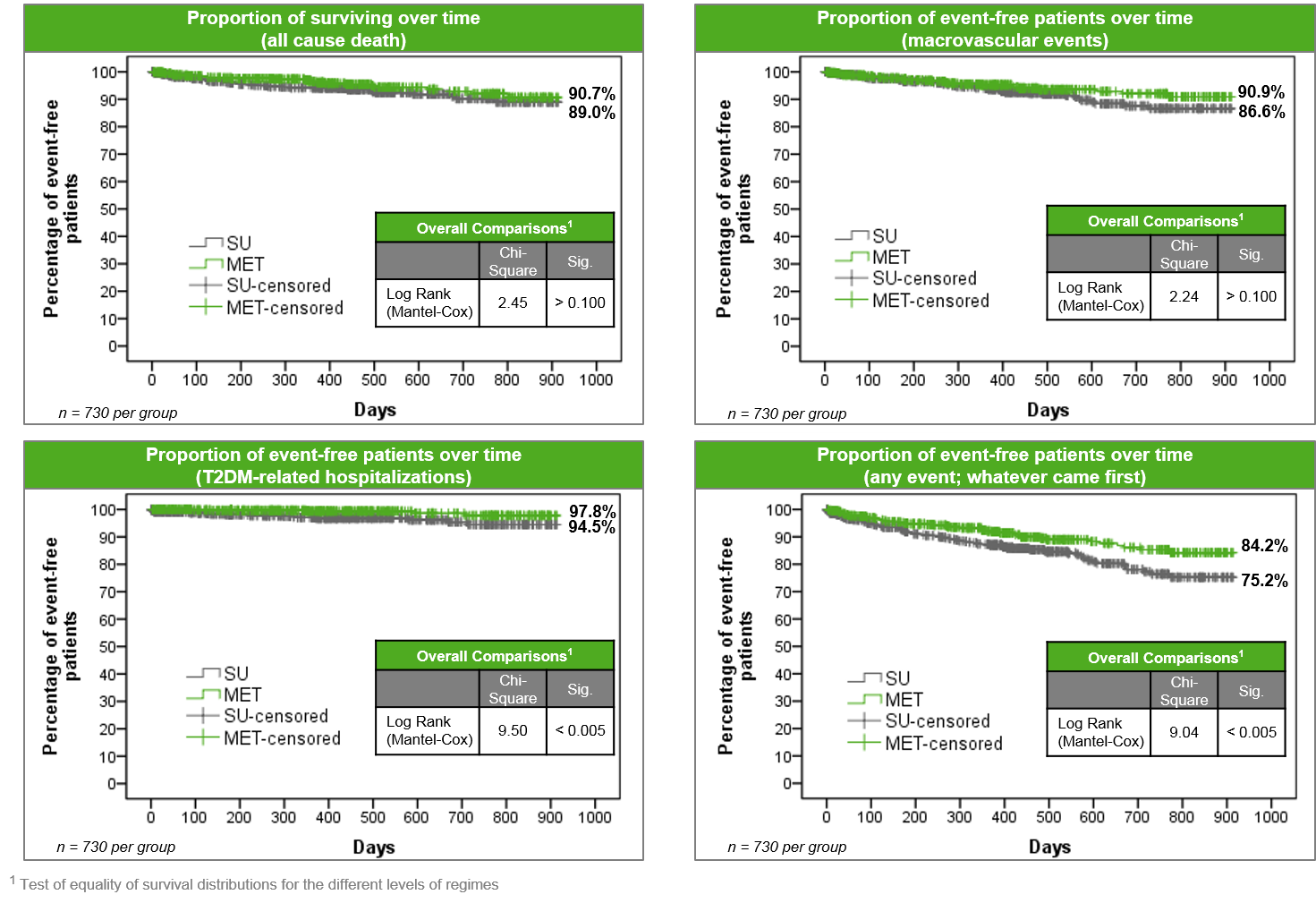

Supplement: Supplementary file 6 — Kaplan-Meier (KM) curves for all-cause death rates, macrovascular event rates and T2DM-related hospitalizations for patients with either MET or SU monotherapy (PS matched groups). The figure shows KM curves representing the percentage of event-free patients (all-cause event as well as mortality, MACE and T2DM-related hospitalizations) for two T2DM-incident cohorts: patients who received SU monotherapy and patients who received MET monotherapy. Cohorts are matched by PSM. Observation started with the first observed SU/MET prescription. (TIF 546 kb) [file 40200_2016_251_MOESM6_ESM.tif]

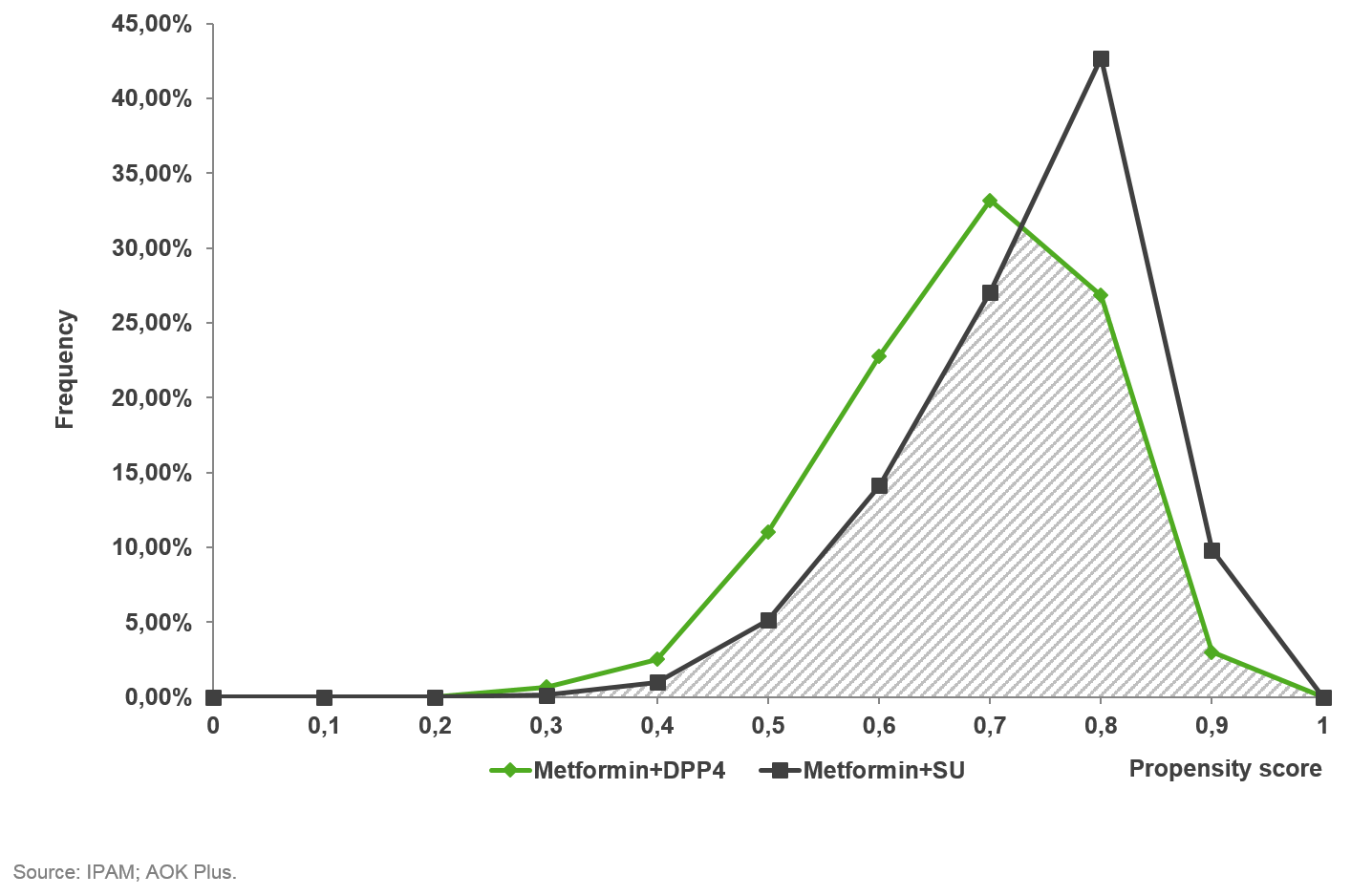

Supplement: Supplementary file 7 — Distribution of propensity scores as calculated by logistic regression for SU+MET and DPP4-MET combination therapy users. This figure describes the overlap of propensity scores in Cohort 2, incorporating patients who received SU+MET or DPP4-MET combination therapy. (TIF 227 kb) [file 40200_2016_251_MOESM7_ESM.tif]

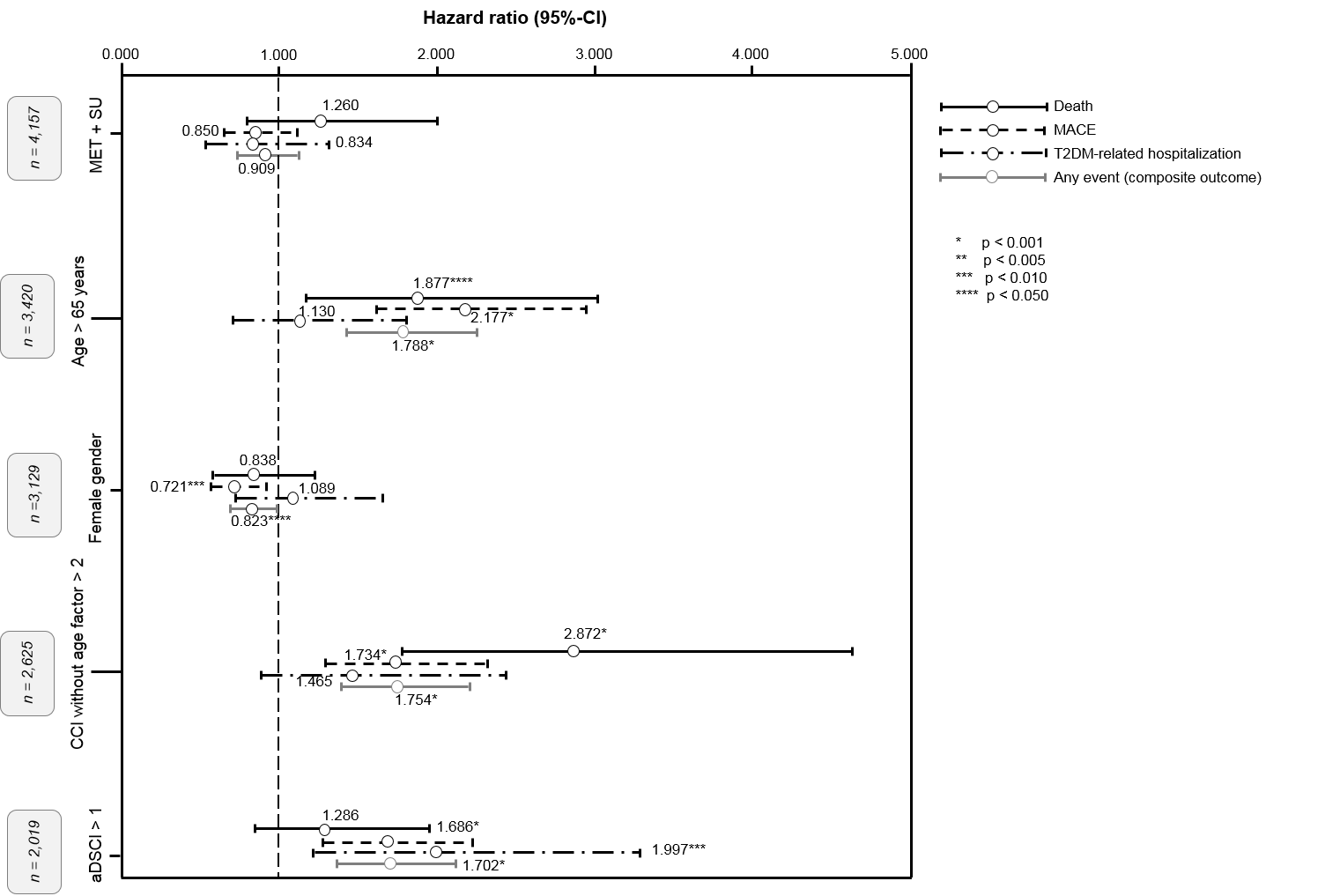

Supplement: Supplementary file 8 — Multivariable Cox regression models estimating time to event for four outcome categories (MET+SU/MET+DPP-4 therapy). Factors associated with event risk. The figure shows the results of the multivariable Cox regression analysis with regard to independent factors influencing time until an event (all-cause event as well as mortality, MACE and T2DM-related hospitalizations in separate models) in the T2DM-prevalent sample that received either SU+MET or DPP4+MET combination therapy. (TIF 160 kb) [file 40200_2016_251_MOESM8_ESM.tif]

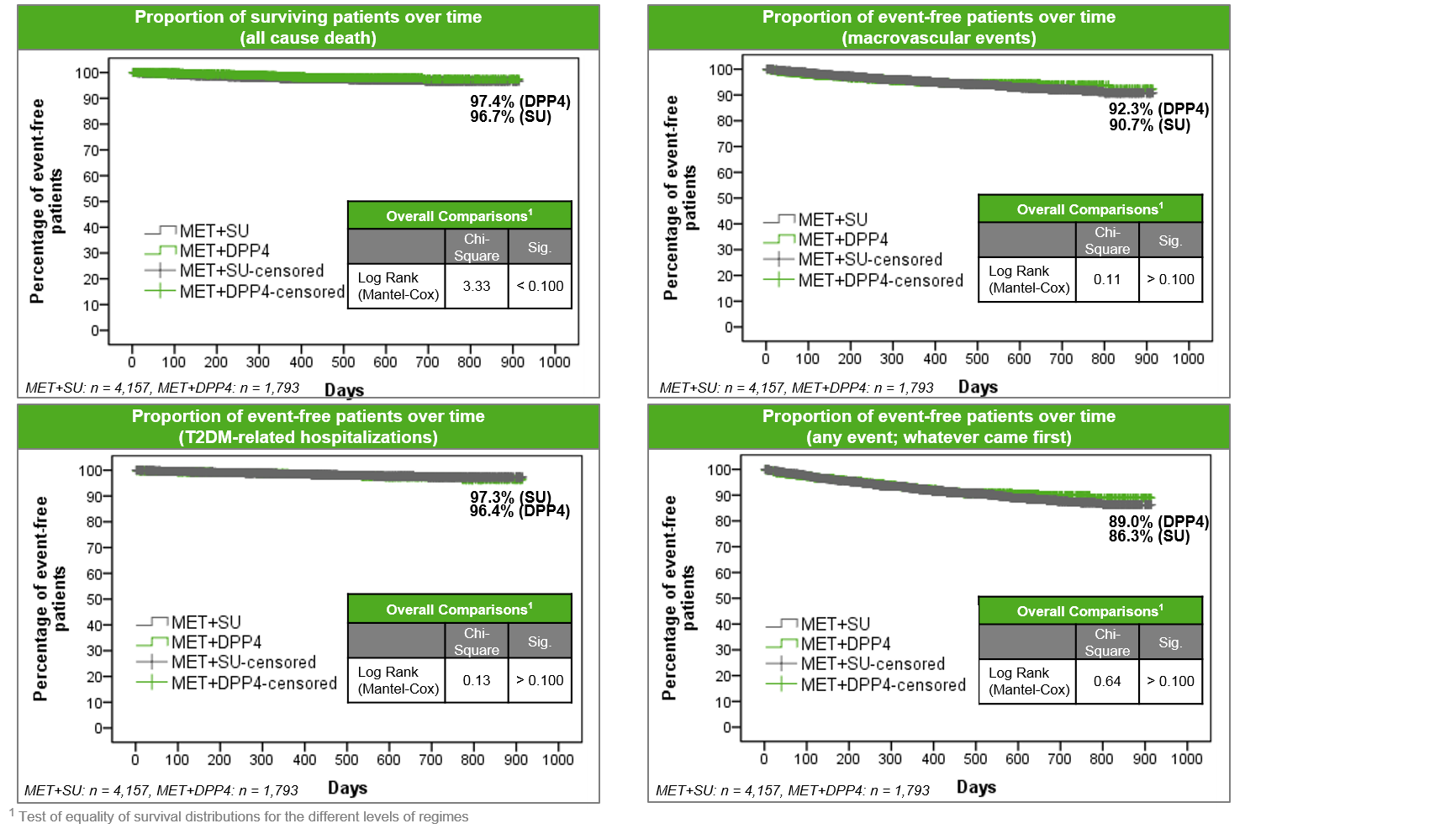

Supplement: Supplementary file 9 — Kaplan-Meier (KM) curves for crude all-cause death rates, macrovascular event rates and T2DM-related hospitalizations for patients with either MET+SU or MET+DPP-4 therapy. The figure shows KM curves representing the percentage of event-free patients (all-cause event as well as mortality, MACE and T2DM-related hospitalizations) for the two cohorts defined above. Observation started with the first observed prescription of the second combination agent. (TIF 604 kb) [file 40200_2016_251_MOESM9_ESM.tif]

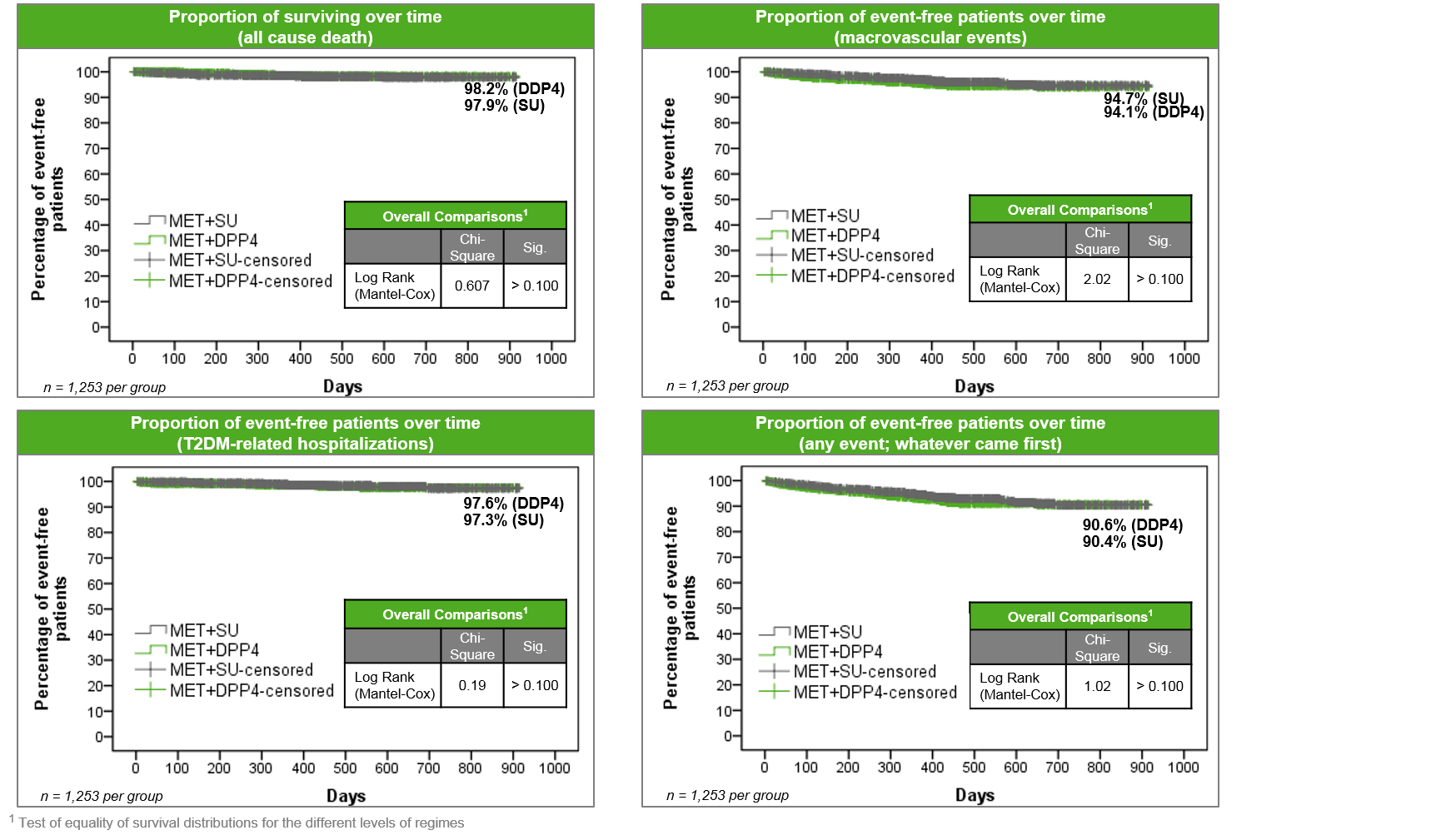

Supplement: Supplementary file 10 — Kaplan-Meier (KM) curves for crude all-cause death rates, macrovascular event rates and T2DM-related hospitalizations for patients with either MET+SU or MET+DPP-4 therapy (PS matched groups). The figure shows KM curves representing the percentage of event-free patients (all-cause event as well as mortality, MACE and T2DM-related hospitalizations) for the two cohorts defined above. Observation started with the first observed prescription of the second combination agent. (TIF 629 kb) [file 40200_2016_251_MOESM10_ESM.tif]
